# Li_5_SnP_3_ – a Member of the Series Li_10+4*x*_Sn_2−*x*_P_6_ for *x*=0 Comprising the Fast Lithium‐Ion Conductors Li_8_SnP_4_ (*x*=0.5) and Li_14_SnP_6_ (*x*=1)

**DOI:** 10.1002/chem.202104219

**Published:** 2022-01-27

**Authors:** Stefan Strangmüller, David Müller, Gabriele Raudaschl‐Sieber, Holger Kirchhain, Leo van Wüllen, Thomas F. Fässler

**Affiliations:** ^1^ Department of Chemistry Technische Universität München Lichtenbergstraße 4 85747 Garching bei München Germany; ^2^ Department of Chemistry Chair of Inorganic and Metal-Organic Chemistry Technical University of Munich Lichtenbergstraße 4 85747 Garching bei München Germany; ^3^ Department of Physics University of Augsburg Universitätsstraße 1 86159 Augsburg Germany

**Keywords:** ball milling, lithium-ion conductors, NMR spectroscopy, phosphidotetrelates, X-ray diffraction

## Abstract

The targeted search for suitable solid‐state ionic conductors requires a certain understanding of the conduction mechanism and the correlation of the structures and the resulting properties of the material. Thus, the investigation of various ionic conductors with respect to their structural composition is crucial for the design of next‐generation materials as demanded. We report here on Li_5_SnP_3_ which completes with *x*=0 the series Li_10+4*x*_Sn_2−*x*_P_6_ of the fast lithium‐ion conductors *α*‐ and *β*‐Li_8_SnP_4_ (*x*=0.5) and Li_14_SnP_6_ (*x*=1). Synthesis, crystal structure determination by single‐crystal and powder X‐ray diffraction methods, as well as ^6^Li, ^31^P and ^119^Sn MAS NMR and temperature‐dependent ^7^Li NMR spectroscopy together with electrochemical impedance studies are reported. The correlation between the ionic conductivity and the occupation of octahedral and tetrahedral sites in a close‐packed array of P atoms in the series of compounds is discussed. We conclude from this series that in order to receive fast ion conductors a partial occupation of the octahedral vacancies seems to be crucial.

## Graphical abstract

**The compound Li_5_SnP_3_** completes for x=0 the series Li_10+4*x*_Sn_2−*x*_P_6_ (*x*=0–1) of lithium‐ion conducting phosphidostannates. The thorough investigation of the crystal structure as well as the materials properties applying X‐ray diffraction as well as NMR and electrochemical impedance spectroscopy allows for a detailed comparison of the materials structure‐property relationships, and thus, improves the understanding of the conduction mechanism within solid ion conductors.

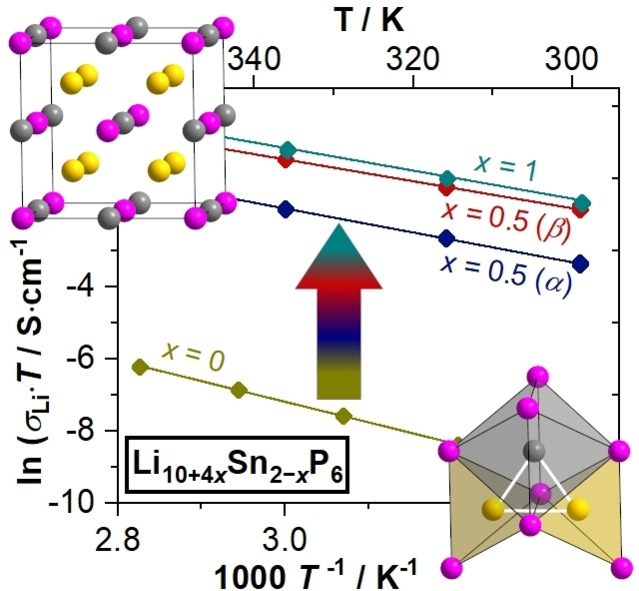

## Introduction

Solid‐state electrolytes (SE) are predicted to dominate mainly in electric vehicles and future lithium battery chemistry.^[1]^ Therefore, extensive efforts are made aiming for the discovery of SE materials that are suitable to meet demanded properties for application in all‐solid‐state batteries.[^2^, ^3^, ^4^] Another approach focuses on a better understanding of the origin of materials’ properties, such as ionic conductivity. The elaboration of structure‐property relationships by comparison of a variety of crystalline candidate materials that comprise diverse structural differences with respect to their electronic properties allows for designing and tailoring of materials’ properties as demanded.[^5^, ^6^, ^7^, ^8^] Searching for high‐performance ionic conductors, a large number of innovative SEs featuring ever increasing ionic conductivities has been reported over the last decades.[^2^, ^3^, ^4^, ^9^, ^10^] But a thorough investigation of structure‐property relationships also demands the evaluation of less powerful materials in order to unveil the reasons for favorable or unfavorable properties.

The recently introduced family of lithium phosphidotetrelates and the closely related lithium phosphidotrielates are well‐suited for further analyses of ionic conduction mechanisms as this class of materials offers a broad structural variety as well as a corresponding large variety of properties. For example, several compounds with a fast ionic conduction of up to 3×10^−3^ S cm^−1^ have been reported[^3^, ^4^, ^7^] next to materials that feature a band gap of about 3 eV, indicative of semiconducting behavior.^[11]^ In addition the compound LiGe_3_P_3_ shows moderate electric conductivity and an unprecedented stability when exposed to water and air.^[12]^ Apart from the latter and a few other exceptions, most of the so far discovered lithium phosphidotetrelates and ‐trielates are based on tetrahedral [*Tt*P_4_] or [*Tr*P_4_] units, which occur either as isolated [*Tt*P_4_]^8−^ or [*Tr*P_4_]^9−^ anions that are charge‐compensated by the corresponding amount of Li^+^ or they build frameworks of condensed tetrahedra and supertetrahedra, respectively.[^8^, ^11^, ^12^, ^13^, ^14^, ^15^]

The compound Li_10_Si_2_P_6_ comprises pairs of edge‐sharing [SiP_4_] units resulting in the polyanion [Si_2_P_6_]^10−^.^[16]^ Due to these building blocks, the formula is commonly given as Li_10_Si_2_P_6_ rather than Li_5_SiP_3_ to express the molecular‐anionic character. Indeed, a compound with the composition Li_5_SiP_3_ has been reported in the 1950s, and characterized by the determination of the cubic space group *Fm* $\bar{3}$ *m* with a lattice parameter of *a*=5.852 Å.^[17]^ The structure is closely related to the antifluorite type or a defect variant of the Li_3_Bi structure,^[18]^ which can be described by a *ccp* of P atoms in which all tetrahedral voids are statistically occupied by Li^+^ and Si^4+^ in a mixing ratio of 5 : 1. So far, all attempts to reproduce these findings have failed.[^13^, ^16^] However, about 20 years later the same structure was reported for the heavier homologue Li_5_SnP_3_ ^[19]^ with identical cubic space group *Fm* $\bar{3}$ *m* (no. 225) and a lattice parameter of *a*=5.97 Å. Further investigations of the material's properties are still pending, whereas the lithium‐rich phosphidostannates *α*‐ and *β*‐Li_8_SnP_4_ as well as Li_14_SnP_6_ have been recently reported to show superionic lithium‐ion conductivities of about 1×10^−3^ S cm^−1^. Interestingly in *α*‐ and *β*‐Li_8_SnP_4_, the Li and Sn atoms are fully ordered in the tetrahedral voids of the *ccp* of P atoms, thus leading to polyanionic SnP_4_^8−^ units. By contrast in Li_14_SnP_6_, the Li and Sn atoms are statistically distributed over the tetrahedral sites. Analysis of Li^+^ diffusion pathways based on powder neutron diffraction data unveiled structural variations, which were directly connected to the different values of the ionic conductivities of the three compounds.[^7^, ^8^]

In the following we report on the systematic investigation of the system Li_10+4*x*_Sn_2−*x*_P_6_ (*x*=0.0 to 1.0) including the compounds Li_5_SnP_3_, (*α*‐ and *β*‐) Li_8_SnP_4_ and Li_14_SnP_6_ that arise for *x*=0.0, 0.5, and 1.0, respectively. Following a well‐established synthesis route for lithium phosphidotetrelates including mechano‐chemical milling allows for the first time the isolation and detailed characterization of the compound Li_5_SnP_3_ by single‐crystal and powder X‐ray diffraction data completed by Rietveld refinement as well as ^6^Li, ^31^P and ^119^Sn solid‐state magic angle spinning (MAS) NMR measurements. Differential scanning calorimetry (DSC) and isothermal annealing experiments of the reactive mixtures obtained via mechanical alloying were carried out to investigate the thermal properties of the materials. Furthermore, the Li^+^ mobility and its activation energy, as well as the ionic and electronic conductivity were determined via temperature‐dependent ^7^Li NMR spectroscopy and electrochemical impedance spectroscopy (EIS). Finally, all data and the associated properties are compared to that of the recently reported lithium phosphidostannates *α*‐ and *β*‐Li_8_SnP_4_ and Li_14_SnP_6_, which allows for the formulation of new structure‐property relationships regarding the ionic conductivity in solid‐state Li^+^ conductors.

## Experimental Section

All syntheses were carried out under Ar atmosphere in glove boxes (MBraun, 200B) with moisture and oxygen levels below 0.1 ppm, or in containers, which were sealed under Ar atmosphere and vacuum (<2 ⋅ 10^−2^ mbar), respectively. Lithium phosphidostannates are sensitive to oxygen and moisture; in particular, contact with water results in a vigorous reaction including the formation of flammable and toxic gases (e. g., phosphine). Therefore, disposal must be addressed in small amounts at a time and under proper ventilation.

**Bulk Synthesis via Ball Milling and Annealing**: All samples were prepared by a well‐established synthesis route starting from the elements, lithium (Rockwood Lithium, 99 %), tin (Merck, 99.9 %) and red phosphorus (ChemPUR, 99.999 %) in stoichiometric amounts aiming for compositions according to the formula Li_10+4*x*_Sn_2−*x*_P_6_ with *x*=0.00, 0.25, 0.50, 0.75, 1.00 (Table 1), followed by annealing at moderate temperatures.

**Table 1:** Overview of the prepared “reactive mixtures” according to the formula Li_10+4*x*_Sn_2−*x*_P_6_ (*x*=0.0 to 1.0).

*x*	Composition
0.00	Li_10_Sn_2_P_6_=Li_5_SnP_3_
0.25	Li_11_Sn_1.75_P_6_
0.50	Li_12_Sn_1.5_P_6_=Li_8_SnP_4_
0.75	Li_13_Sn_1.25_P_6_
1.00	Li_14_SnP_6_

In the first step, a “reactive mixture” (*m*=5.0 g) was prepared by mechano‐chemical milling using a Retsch PM100 Planetary Ball Mill (350 rpm, 18 h, 10 min interval, 3 min break) with a tungsten carbide milling jar (*V*=50 mL) and three balls with a diameter of 15 mm.

In the second step, the “reactive mixture” was pressed into pellets, sealed in batches of 0.3 to 1.0 g in carbon‐coated silica glass ampules and heated in a muffle furnace (Nabertherm, L5/11/P330) to 673, 773 or 973 K (heating rate: 4 K min^−1^) for 24 h, followed by quenching of the hot ampules in water.

**Powder X‐Ray Diffraction and Rietveld Refinement**: Data were collected at room temperature on a STOE Stadi P diffractometer (Ge(111) monochromator, Cu_*Kα*1_ radiation, *λ*=1.54056 Å or Mo_*Kα*1_ radiation, *λ*=0.70932 Å) with a Dectris MYTHEN 1 K detector in Debye‐Scherrer geometry. Samples were sealed in glass capillaries (Ø 0.3 mm) for measurement. Raw data were processed with the WinXPOW^[20]^ software prior to refinement.

The data analysis of Li_5_SnP_3_ was performed using the full profile Rietveld method implemented in the FullProf program package.^[21]^ To model the peak profile, the pseudo‐Voigt function was chosen. The background contribution was determined using a linear interpolation between selected data points in non‐overlapping regions. The scale factor, zero angular shift, profile shape parameters, resolution (Caglioti) parameters, asymmetry and lattice parameters as well as fractional coordinates of atoms and their displacement parameters were varied during the fitting. Free refinement of the occupancy of the 8*c* site by Sn and Li exhibited only marginal deviations from the electron‐precise formula Li_5_SnP_3_ (*Z*=1.33) or Li_6.67_Sn_1.33_P_4_ (*Z*=1). The corresponding data are given as Supporting Information. In addition, a second refinement was carried out with site occupancies set to the exact stoichiometry. Since the results of both refinements were in very good agreement, the electron precise stoichiometry Li_5_SnP_3_ is assumed. All structures were visualized using DIAMOND.^[22]^

**Synthesis of powdery and single‐crystalline Li_5_SnP_3_**: Li_5_SnP_3_ is obtained as black powder on a gram scale and in high purity by annealing of the “reactive mixture” of the nominal composition “Li_5_SnP_3_” (Li_10+4*x*_Sn_2−*x*_P_6_ with *x*=0.0) in carbon‐coated silica glass ampules at 773 K for 24 h, followed by quenching of the hot ampule in water. The weight fraction of remaining *β*‐Sn was determined via Rietveld refinement to 0.8(1) %.

Single crystals were obtained by a high‐temperature reaction of lithium (Rockwood Lithium, 99 %), tin (Merck, 99.9 %) and red phosphorus (Sigma‐Aldrich, 97 %) in a ratio corresponding to “Li_5_SnP_6_”. The elements were annealed for 18 h at 873 K (heating rate: 4 K min^−1^) in a sealed tantalum ampule and subsequently quenched in water.

**Single‐crystal X‐ray Diffraction Data Collection**: A single crystal of Li_5_SnP_3_ was isolated and sealed in a glass capillary (0.1 mm). For diffraction data collection, the capillary was positioned in a 150 K cold N_2_ gas stream. Data collection was performed with a STOE StadiVari (Mo_Kα1_ radiation) diffractometer equipped with a DECTRIS PILATUS 300 K detector. Structures were solved by Direct Methods (SHELXS‐2014) and refined by full‐matrix least‐squares calculations against *F* ^2^ (SHELXL‐2014).^[23]^

Further details of the crystal structure investigations may be obtained from the joint CCDC/FIZ Karlsruhe online deposition service: Deposition Number(s) CSD‐2074706 (Li_6.6667_Sn_1.33_P_4_, single crystal), CSD‐2074707 (Li_6.6667_Sn_1.33_P_4_, powder), CSD‐2074709 (Li_6.74_Sn_1.29_P_4_, single crystal), and CSD‐2074710 (Li_6.70_Sn_1.30_P_4_, powder) contain(s) the supplementary crystallographic data for this paper. These data are provided free of charge by the joint Cambridge Crystallographic Data Centre and Fachinformationszentrum Karlsruhe Access Structures service.

**Differential Scanning Calorimetry (DSC)**: The thermal behavior of the compounds was studied with a Netzsch DSC 404 Pegasus device. Niobium crucibles were filled with the samples and sealed by arc‐welding. Empty sealed crucibles served as a reference. Measurements were performed under an Ar flow of 75 mL min^−1^ and a heating/cooling rate of 10 K min^−1^. Data collection and handling were carried out with the Proteus Thermal Analysis program,^[24]^ and visualization was realized using OriginPro 2020.^[25]^

**Solid‐State NMR Spectroscopy**: Magic angle spinning (MAS) NMR spectra were recorded on a Bruker Avance 300 NMR device operating at 7.04 T by the use of a 4 mm ZrO_2_ rotor. The resonance frequencies of the measured nuclei are 44.2, 121.5 and 111.9 MHz for ^6^Li, ^31^P and ^119^Sn, respectively. The rotational frequency was set to 15 kHz. The MAS spectra were acquired at room temperature with recycle delays of 10 to 30 s and 1000 to 2736 scans. All ^6^Li spectra were referenced to LiCl (1 m, aq) and LiCl (s) offering chemical shifts of 0.0 ppm and −1.15 ppm, respectively. The ^31^P spectra were referred to (NH_4_)H_2_PO_4_(s) (ammonium dihydrogen phosphate) with a chemical shift of 1.11 ppm with respect to concentrated H_3_PO_4_ (aq) (phosphoric acid). SnO_2_ (s) (Cassiterite) was used as a secondary standard for the ^119^Sn spectra, showing a chemical shift of −604.3 ppm[^26^, ^27^] referred to (CH_3_)_4_Sn(l) (tetramethylstannane). All spectra were recorded using single‐pulse excitation.

Static ^7^Li NMR experiments were performed using a Bruker Avance III spectrometer operating at a magnetic field of 7 T employing a 4 mm WVT MAS probe. The resonance frequency of the ^7^Li nucleus is 116.6 MHz. The sample was sealed in a 4 mm glass tube to avoid contact with air and moisture. The temperature calibration for the measurements was performed using the temperature‐dependent ^207^Pb NMR shift of lead nitrate (Pb(NO_3_)_2_) as chemical shift thermometer, which was also measured in a sealed glass tube. A saturation comb had been used prior to the ^7^Li data acquisition. The spectra were recorded in the temperature range from room temperature to 200 K with recycle delays of 60 s and 4 scans. All spectra were referenced to LiCl (9.7 M, aq).

**Impedance Spectroscopy and DC Conductivity Measurements**: Potentiostatic impedance spectroscopy was carried out using a Biologic SP‐300 potentiostat in a frequency range of 7 MHz to 100 mHz with an excitation amplitude of ±10 mV. All measurements were conducted in an argon‐filled glove box. Powder samples of Li_5_SnP_3_ (300 mg) were measured in a custom‐built symmetric cell (Ø=8 mm) with hardened steel electrodes under blocking conditions. Pressure was applied by six M14 screws, fastened with a defined torque of 30 Nm each, translating to proximately 480 MPa, so that the sample was compressed to 88 % of its crystallographic density. A more detailed description can be found in the literature.^[3]^ The temperature was controlled via a Julabo Dyneo DD 1000 Thermostat feeding an aluminum heating block, which enclosed the measurement cell. The electric conductivity was measured in the same cell setup with three polarization steps of 50, 100 and 150 mV, each held for 6 h to ensure equilibrium conditions.

## Results

### Syntheses

For the systematic investigation of lithium‐rich ternary lithium phosphidostannates, mixtures with nominal compositions according to Table 1 were alloyed mechanically in a ball mill. The compositions were chosen according to the formula Li_10+4*x*_Sn_2−*x*_P_6_ (*x*=0.0 to 1.0) including also the compounds Li_5_SnP_3_,^[19]^ *α*‐ and *β*‐Li_8_SnP_4_,^[8]^ as well as Li_14_SnP_6_ ^[7]^ for *x*=0.0, 0.5 and 1.0, respectively.

In order to detect further phases within this family of materials and to reveal existing phase widths of the compounds, the “reactive mixtures” were annealed at 673, 773 and 973 K, respectively. All “reactive mixtures” and products were analyzed using powder X‐ray diffraction (PXRD) data for the identification of the occurring phases within the samples as well as for the determination of the cell parameters of the lithium phosphidostannates. The data showed the formation of the known compounds Li_5_SnP_3_,^[19]^ *α*‐ and *β*‐Li_8_SnP_4_ ^[8]^ and Li_14_SnP_6_ ^[7]^ as well as the presence of Li_3_P and remaining *β*‐Sn. Consequently, no phase widths were observed for these compounds. Details of the results and all PXRD patterns are given as Supporting Information.

**Figure 1:**
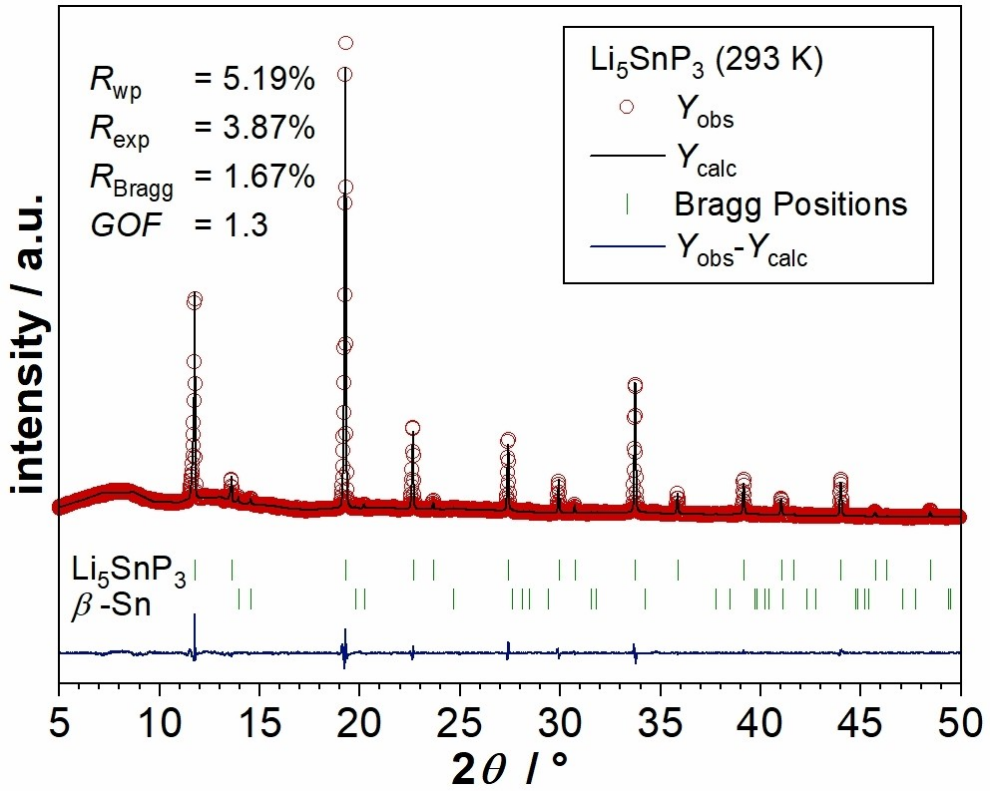
Results of the Rietveld analysis of the powder X‐ray diffraction pattern of Li_5_SnP_3_ at 293 K. Red circles indicate observed intensities *Y*_obs_, black lines show calculated intensities *Y*_calc_, blue lines reveal the difference between observed and calculated intensities, and green marks indicate Bragg positions of Li_5_SnP_3_ (weight fraction 99(1) %) and *β*‐Sn (weight fraction 0.8(1) %), respectively.

For the structural reinvestigation of Li_5_SnP_3_ the corresponding “reactive mixture” obtained by ball milling of the elements in stoichiometric amounts was annealed at 773 K for 24 h, followed by quenching of the hot ampule in water. By this method, the material is accessible on a gram scale and in high purity as indicated by Rietveld analysis (Figure 1). Details of the refinement are shown in Table 2.

**Table 2:** Details of the Rietveld structure refinements of Li_5_SnP_3_ (*Z*=1.33) at 293 K.

Empirical formula	Li_6.67_Sn_1.33_P_4_
*T* [K]	293
formula weight [g mol^−1^]	328.08
Group (no.)	*Fm* $\bar{3}$ *m* (225)
Unit cell parameters [Å]	*a*=5.98715(5)
*Z*	1
*V* [Å^3^]	214.615(3)
*ρ*_calc._ [g cm^−3^]	2.541
*2θ* range [deg]	5.000–49.9441
*R*_p_	3.87 %
*R*_wp_	5.19 %
*R*_exp_	3.87 %
*Χ* ^2^	1.80
*GOF*	1.3
*R*_Bragg_	1.67 %
*R*_f_	1.48 %
Depository no.	CSD‐2074707

Differential scanning calorimetry, followed by PXRD measurements of the samples indicate the decomposition of Li_5_SnP_3_ at high temperatures, resulting in a mixture of *β*‐Sn and another cubic phase, indicated by additional reflections assignable to a superstructure as observed for the ordered structures of *α*‐ and/or *β*‐Li_8_SnP_4_. Since the additional reflections are broadened, a partial ordering of the cations is assumed. The corresponding thermograms and PXRD patterns as well as a detailed discussion of the results is given as Supporting Information.

**Figure 2:**
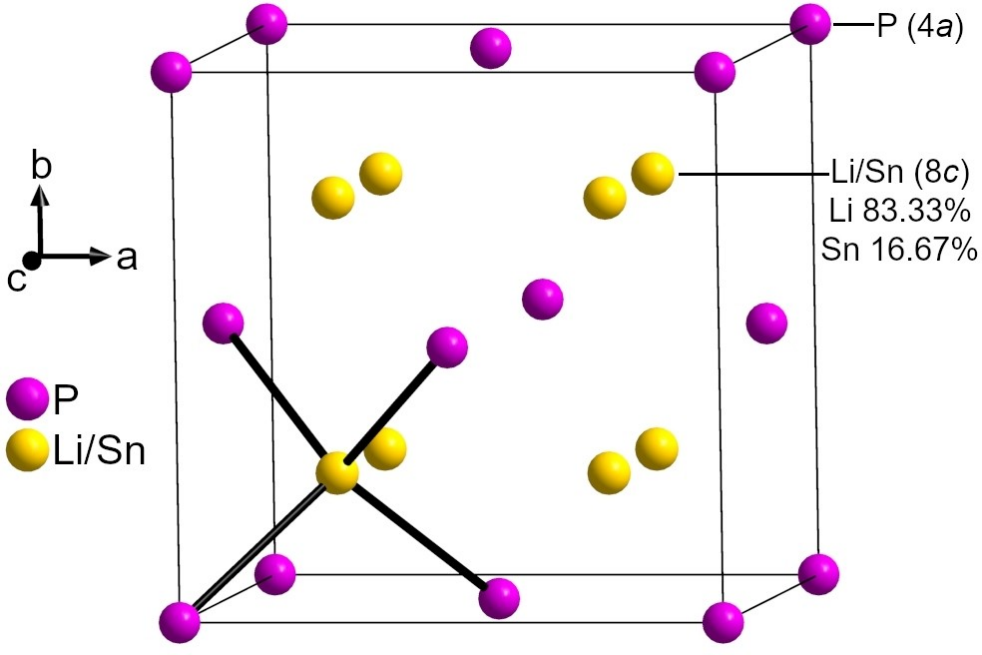
Structure of Li_5_SnP_3_ from single‐crystal data at 150 K. P atoms (4*a*), and mixed Li/Sn sites (8*c*, Li 83.33 % and Sn 16.67 %) are depicted as pink and gold displacement ellipsoids, respectively, all set at 90 % probability. Black lines mark (Li/Sn)‐P bonds, resulting in (Li/Sn)P_4_ tetrahedra.

In accordance with previous reports,^[19]^ the single‐crystal data of Li_5_SnP_3_ indicate the cubic space group *Fm* $\bar{3}$ *m* (no. 225) and a lattice parameter of *a*=5.9541(7) Å at 150 K (Figure 2 and Table 3).

**Table 3:** Crystallographic data and refinement parameters of Li_5_SnP_3_ (*Z*=1.33) or Li_6.67_Sn_1.33_P_4_ (*Z*=1) at 150 K with fixed site occupancy factors.

empirical formula	Li_5_SnP_3_/Li_6.67_Sn_1.33_P_4_
Formula weight [g mol^−1^]	328.08
Crystal size [mm^3^]	0.08×0.08×0.09
Crystal color	black
*T* [K]	150
Crystal system	cubic
Space group (no.)	*Fm* $\bar{3}$ *m* (225)
Unit cell parameter [Å]	*a*=5.9541(7)
*Z*	0.75/1
*V* [Å^3^]	211.08(7)
*ρ*_*calc*._ [g cm^−3^]	2.583
*μ* [mm^−1^]	4.644
*F*(000) [e]	147
*θ* range [deg]	5.934–46.355
Index range (*hkl*)	−7≤*h*≤11, −10≤*k*≤11, −11≤*l*≤4
Reflections collected	348
Independent reflections	72
*R_int_*	0.0101
Reflections with *I*>2*σ*(*I*)	72
Absorption correction	multi‐scan
Data/restraints/parameters	72/0/4
Goodness‐of‐fit on *F* ^2^	1.248
*R*_1_, *wR*_2_ (all data)	0.0231, 0.0231
*R*_1_, *wR*_2_ [*I*>2*σ*(*I*)]	0.0626, 0.0626
Largest diff. peak and hole [e Å^−3^]	0.968/−0.497
Depository no.	CSD‐2074706

The structure of Li_5_SnP_3_ can be described as a *ccp* of P atoms (4*a* site) with the Sn and Li atoms statistically distributed in all tetrahedral voids (8*c* site) with a Sn : Li ratio of 1 : 5. The structure is thus closely related to the antifluorite structure with P and Li/Sn on Ca and F atom positions, respectively. The structure was also confirmed by powder X‐ray diffraction and Rietveld refinement at 293 K. Atomic coordinates and anisotropic displacement parameters as well as the results from the powder X‐ray diffraction at 293 K and the single‐crystal X‐ray diffraction at 150 K are given in the Supporting Information.

The structure of the lithium‐rich compound Li_14_SnP_6_ is almost isotypic to that of Li_5_SnP_3_, but with a slightly larger lattice parameter (*a*=6.01751(3) Å) and a different occupation of the mixed Li/Sn positions in the tetrahedral voids and – due to the higher Li amount – partially occupied octahedral sites (4*b*). The same Li_3_Bi‐type structure was also observed for the lighter homologues Li_14_SiP_6_ and Li_14_GeP_6_.[^3^, ^7^]

All interatomic Li/Sn−P (2.5782(2) Å), Li/Sn−Li/Sn (2.9771(3) Å) and P−P distances (4.2102(3) Å) are within the range of those found for related ternary or binary compounds like Li_14_ *Tt*P_6_ (*Tt*=Si, Ge, Sn),[^3^, ^7^] (*α*‐/*β*‐)Li_8_ *Tt*P_4_ (*Tt*=Si, Ge, Sn)[^8^, ^13^, ^14^] and Li_3_P.^[28]^

**Figure 3:**
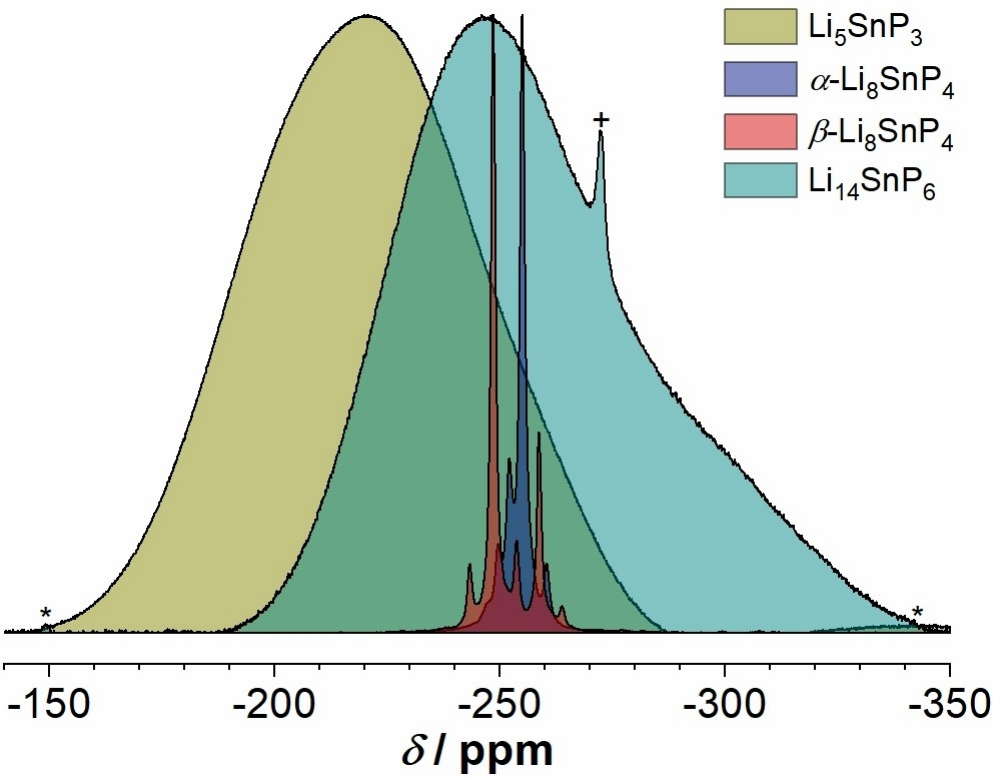
Overview of the ^31^P MAS NMR spectra of Li_5_SnP_3_ (olive), *α*‐Li_8_SnP_4_ (blue), *β*‐Li_8_SnP_4_ (red),^[8]^ and Li_14_SnP_6_ (teal).^[7]^ Spinning sidebands and Li_3_P (impurity) are indicated by * and +, respectively.

The ^31^P MAS NMR spectrum of Li_5_SnP_3_ shows one very broad resonance (∼17 kHz) at a chemical shift of −220.3 ppm (Figure S8). A comparable broadening was also observed in case of the structurally related and highly disordered compounds Li_14_ *Tt*P_6_ (*Tt*=Si, Ge, Sn).[^3^, ^7^, ^29^] Furthermore, it is assumed that the in some extend very complex coupling of Sn and P atoms also leads to a merging of signals with a related chemical shift as recently reported for example, for *α*‐ and *β*‐Li_8_SnP_4_.^[8]^ In comparison to the resonances of the latter and of other closely related lithium phosphidostannates such as Li_14_SnP_6_,^[7]^ the maximum of the signal appertaining to Li_5_SnP_3_ shows a downfield shift of about 20 to 40 ppm. This indicates a lower shielding of the P atoms and hints for a lower formal charge (<−2) and to a higher coordination number of P by Sn atoms (Figure 3). At the local level, all P atoms in Li_5_SnP_3_ are covalently bound to at least one Sn atom, whereas in Li_14_ *Tt*P_6_ also P^3−^ anions are present according to [(Li^+^)_14_(*Tt*P_4_)^8−^(P^3−^)_2_]. In analogy to the structures of *α*‐ and *β*‐Li_8_SnP_4_ and Li_14_SnP_6_, respectively, the Sn atoms are occupying tetrahedral voids, resulting in SnP_4_ units.[^7^, ^8^] Regarding the electron‐precise stoichiometry (Li_5_SnP_3_ or Li_6.67_Sn_1.33_P_4_), each P atom is statistically coordinated by 1.33 Sn atoms. Thus, one would expect the coordination of 1/3 of all P atoms by two Sn atoms, and all the others coordinate to one Sn atom. In other words, two Sn atoms occupy adjacent tetrahedral voids and form edge‐sharing tetrahedra with the formula [Sn_2_P_6_]^10−^. Such units are also observed in the homologous lithium phosphidosilicate Li_10_Si_2_P_6_.^[16]^ However, the [Sn_2_P_6_]^10−^ units are not ordered, and thus, the chemically different P atoms do not appear with a distinct difference in the chemical environment. A further resolution of the broad signal in order to distinguish P atoms located next to only one Sn atom (1b‐P^2−^) and P atoms surrounded by two Sn atoms (2b‐P^1−^) was not feasible. Possible reasons are given after the discussion of the ^119^Sn NMR spectrum.

**Figure 4:**
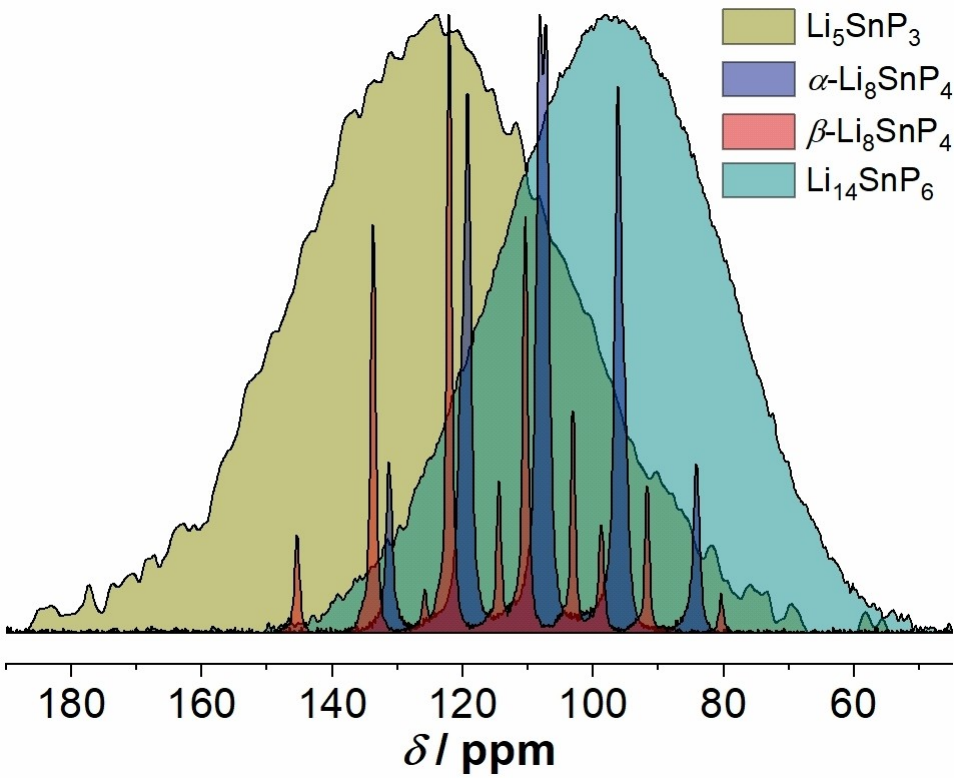
Overview of the ^119^Sn MAS NMR spectra of Li_5_SnP_3_ (olive), *α*‐Li_8_SnP_4_ (blue), *β*‐Li_8_SnP_4_ (red),^[8]^ and Li_14_SnP_6_ (teal).^[7]^

Regarding the ^119^Sn NMR spectrum of Li_5_SnP_3_, the high level of cation disorder also results in only one very broad (∼13 kHz) tin resonance at a chemical shift of 124.6 ppm (Figure S9). In analogy to the ^31^P NMR measurements, this effect has also been observed in the ^119^Sn spectrum of Li_14_SnP_6_.^[7]^ In comparison to the latter, the maximum of the signal for Li_5_SnP_3_ shows a downfield shift of 26.5 ppm, and the resonances of the lithium phosphidostannates Li_5_SnP_3_, *α*‐ and *β*‐Li_8_SnP_4_ and Li_14_SnP_6_ shown in Figure 4 are upfield shifted depending on the Sn to P ratio indicating the slightly different bonding situations within the compounds discussed above.[^7^, ^8^]

Regarding the electron‐precise stoichiometry of the so far known lithium phosphidostannates the structure of Li_14_SnP_6_ shows both, [SnP_4_]^8−^ and P^3−^ units, whereas the two modifications of Li_8_SnP_4_ only contain [SnP_4_]^8−^ tetrahedra, and the basic structure of Li_5_SnP_3_ consists of [Sn_2_P_6_]^10−^ units.[^7^, ^8^] This, in combination with the crystal structures and the just discussed NMR data, at first appears counterintuitively since neither the NMR data nor the crystallographic data clearly resolve the presence of edge‐sharing [SnP_4_] tetrahedra in Li_5_SnP_3_, which are correspondingly reported for Li_10_Si_2_P_6_. In contrast to the strongly covalent character of the Si−P bonds leading to molecule‐like [Si_2_P_6_] units^[16]^ the bonding situation in Li_5_SnP_3_ is assumed to be mainly dominated by the ionic character of the Sn−P bonds resulting in much weaker bonds and, thus, in more uniform chemical environments.

The ^6^Li MAS NMR spectrum shows only one signal corresponding to the one Li site in the structure. The chemical shift of *δ*=4.2 ppm occurs within the characteristic range of ^6^Li resonances reported for lithium phosphidotetrelates and ‐trielates.[^3^, ^4^, ^7^, ^8^, ^11^, ^12^, ^13^, ^14^, ^16^]

### Lithium‐ion mobility

The Li^+^ mobility, the activation energy and the ionic as well as the electronic conductivity are evaluated and compared to recent results of the related compounds *α*‐ and *β*‐Li_8_SnP_4_ and Li_14_SnP_6_.

**Figure 5:**
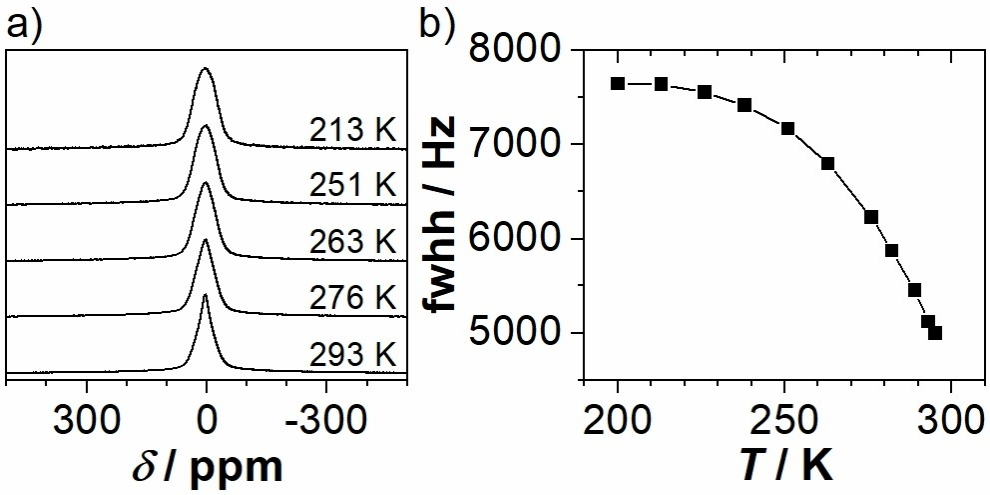
a) Static ^7^Li spectra of Li_5_SnP_3_ at various temperatures; b) evolution of the ^7^Li line width in the temperature range from 200 K to room temperature for Li_5_SnP_3_. The solid line only serves as a guide to the eye.

For a rough estimation of the activation barrier for Li^+^ mobility in crystalline Li_5_SnP_3_ the dynamic behavior of Li^+^ was investigated by temperature‐dependent evolution of the static ^7^Li NMR line width. Since the central transition of the *I*=3/2 ^7^Li nucleus is broadened by homonuclear (^7^Li−^7^Li) and heteronuclear (^7^Li−^31^P) dipolar coupling, both of which scale with the second Legendrian (3 cos^2^ *β*‐1), any dynamic process leads to a (partial) averaging of this orientational dependence and, thus, to a narrowing of the NMR line. The corresponding results are depicted in Figure 5. At 213 K a single Gaussian line was obtained at 3.9 ppm with a line width of about 7.6 kHz. At temperatures above 263 K the signal becomes more heterogeneous and increasingly Lorentz‐shaped, combined with a stronger narrowing of the line. The resonance remains heterogeneous up to 300 K, with a line width of 5.0 kHz. Application of the empirical Waugh‐Fedin relation, $E_{A}^{\mathrm{NMR}}=$ 0.156 ⋅ *T*_onset_ ^[30]^ allows for a rough estimation of the activation energy. Since the high‐temperature plateau is not reached at 300 K an activation energy of $E_{A}^{\mathrm{NMR}}=$ 47 kJ mol^−1^ or higher can be assumed.

In comparison with the corresponding values determined for the lithium‐richer phosphidostannates *α*‐ and *β*‐Li_8_SnP_4_ $(E_{A}^{\mathrm{NMR}}=$ 34 and 28 kJ mol^−1^, respectively) and Li_14_SnP_6_ $(E_{A}^{\mathrm{NMR}}=$ 28 kJ mol^−1^) the estimated activation energy for Li_5_SnP_3_ is by far the highest. Moreover, since the onset temperature *T*_onset_ is estimated to be at 300 K or higher, no or only an extremely low conductivity is expected in electrochemical impedance measurements.

**Figure 6:**
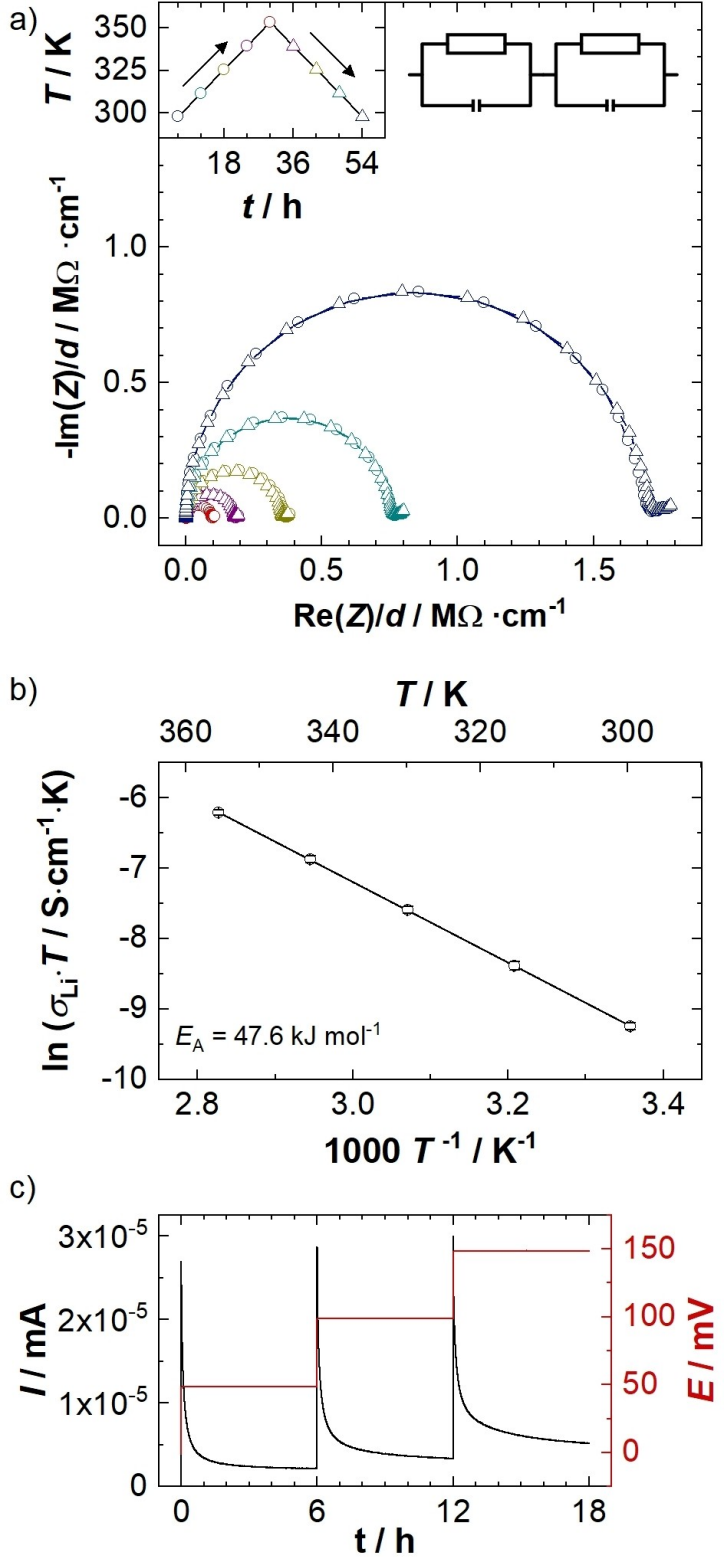
a) Nyquist plot of Li_5_SnP_3_ measured under blocking conditions, with spectra recorded at temperatures between 298 and 353 K according to the color code of the inset. Circles and triangles indicate data collection during heating and cooling, respectively. The equivalent circuit used for fitting is also shown; b) Arrhenius plot of the product of conductivity and temperature (*σ*_Li_ *T*) obtained in one heating‐cooling cycle, with error bars for each temperature based on the standard deviation from independent measurements with three cells; the shown linear fit was used to obtain the activation energy $E_{A}^{\mathrm{PEIS}}$ ; c) polarization curves of Li_5_SnP_3_ for the determination of the electronic conductivity. The black line referring to the left *y* axis shows the recorded current, while the red line (right *y* axis) shows the applied potential steps.

The ionic conductivity of Li_5_SnP_3_ was determined by electrochemical impedance spectroscopy (EIS) in a blocking electrode configuration. The results obtained at temperatures between 298 and 353 K±0.5 K are shown in Figure 6a. The Nyquist plots exhibit well resolved but slightly broadened semicircles and the onset of a branch at low frequencies. For the evaluation of the ionic conductivity only the high‐frequency semicircle was fitted, using two serial R/C elements, revealing two processes involved in the ionic conduction mechanism, that is, a dominant process with a capacity of 3(5) ⋅ 10^−10^ F and a minor process with a capacitance of 5(1) ⋅ 10^−7^ F. The first process can be assigned, according to Irvine et al.,^[31]^ to grain boundary‐controlled ionic conductivity, while the latter resembles the contribution of a surface layer. The overall ionic conductivity at 298 K was determined to 3.2(2) ⋅ 10^−7^ S cm^−1^. Calculated from the slope of the Arrhenius plot in Figure 6b, the activation energy of the ionic mobility was determined to $E_{A}^{\mathrm{PEIS}}=$ 47.6(6) kJ mol^−1^ (∼0.49 eV). The electric conductivity of the sample was studied by polarization of the sample in three different potential steps of 50, 100 and 150 mV, each held until stationary conditions were approached, monitoring the current in the same cell setup as for impedance spectroscopy (Figure 6c). Application of Ohm's law results in an electronic conductivity of 2.1(9) ⋅ 10^−8^ S cm^−1^, which is approximately one order of magnitude lower than the ionic conductivity.

## Discussion and Conclusion

The straightforward synthesis of single crystals and phase‐pure microcrystalline powders finally allows for a comparison of the structure and properties of Li_5_SnP_3_ with that of the recently reported compounds *α*‐ and *β*‐Li_8_SnP_4_ and Li_14_SnP_6_, which contain an increasing percentage of Li^+^.[^7^, ^8^] On both the Li‐poor and Li‐rich sides, mixed Li/Sn positions in a small cubic unit cell occur. The cell parameters shown in Table 4 increase with a higher content of Li^+^ because the exchange of one Sn^4+^ requires the insertion of four Li^+^ to keep the electronic preciseness of the structures. The relatively small amount of Li^+^ in Li_5_SnP_3_ is found to occupy all tetrahedral voids, whereas the octahedral voids remain completely empty, and thus, the octahedral voids must be regarded as energetically less favored. With increasing Li^+^ content, however, also the octahedral voids are progressively filled reaching an occupancy of 25 % in *α*‐ and *β*‐Li_8_SnP_4_ and of 50 % in Li_14_SnP_6_.

**Table 4:** Comparison of the cell parameter *a*, the ionic and electronic conductivities σ_Li_ and σ_el_ and the activation energy $E_{A}^{\mathrm{PEIS}}$ of the lithium phosphidostannates Li_5_SnP_3_, *α*‐ and *β*‐Li_8_SnP_4_ and Li_14_SnP_6_ at ambient temperature.

empirical formula	Li_5_SnP_3_	*α*‐Li_8_SnP_4_	*β*‐Li_8_SnP_4_	Li_14_SnP_6_
*a* [Å] ( $\frac{a}{2}$ [Å])	5.98715	11.97626 (5.98813 Å)	11.99307 (5.996535 Å)	6.01751
*σ*_Li_ [S cm^−1^]	3.2 ⋅ 10^−7^	1.2 ⋅ 10^−4^	6.6 ⋅ 10^−4^	9.3 ⋅ 10^−4^
*σ*_el_ [S cm^−1^]	2.1 ⋅ 10^−8^	1.4 ⋅ 10^−7^	6.1 ⋅ 10^−7^	4.1 ⋅ 10^−7^
$E_{A}^{\mathrm{PEIS}}$ [kJ mol^−1^]	47.6	36.0	32.4	33.8

**Figure 7:**
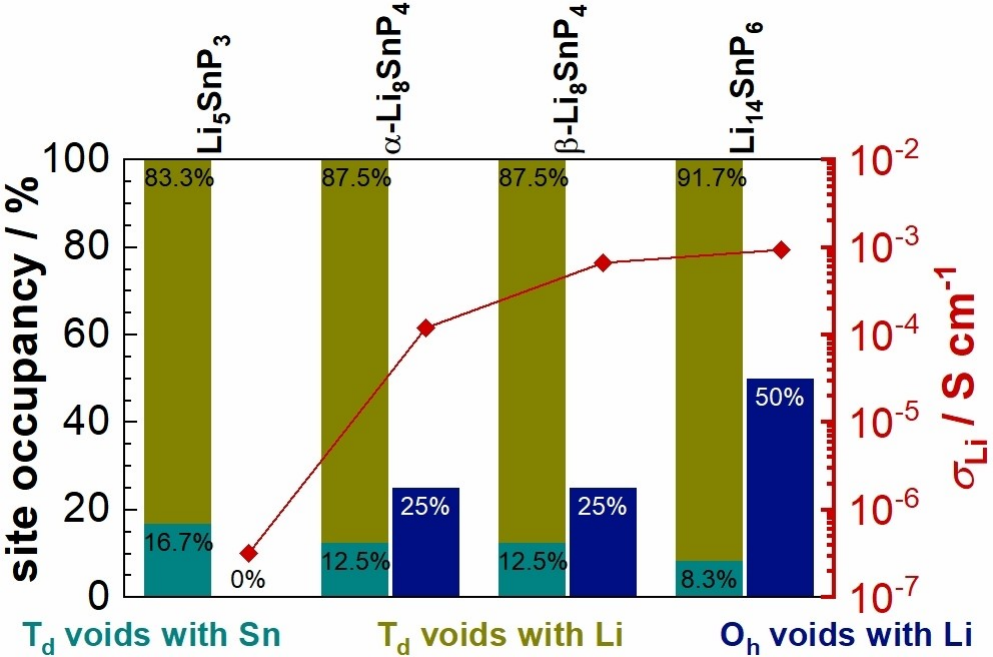
Correlation between the occupation of the tetrahedral and octahedral voids and the resulting ionic conductivity of the phases Li_5_SnP_3_, *α*‐ and *β*‐Li_8_SnP_4_ and Li_14_SnP_6_. The percentages of Sn and Li in the tetrahedral voids are shown in teal and olive, respectively, and the partial occupation of the octahedral voids is indicated in blue. The corresponding ionic conductivity at room temperature is shown in red according to the scale on the right.

In addition, Figure 7 reveals a correlation between the occupancy of the octahedral voids and the ionic conductivity, and the latter increases from Li_5_SnP_3_ to Li_14_SnP_6_ by more than three orders of magnitude. The relatively low ionic conductivity of Li_5_SnP_3_ is attributed to the absence of occupied octahedral voids and corroborates the assumption that these vacancies are energetically less favored. As a consequence, Li^+^ diffusion does not occur via octahedral sites but through edge‐sharing tetrahedral voids that require a higher activation energy if compared to the diffusion along face‐sharing tetrahedral and octahedral voids, as recently shown by the investigation of Li^+^ diffusion pathways in *α*‐ and *β*‐Li_8_SnP_4_ and Li_14_SnP_6_.

The systematic investigation of the ternary Li/Sn/P system within the formula Li_10+4*x*_Sn_2−*x*_P_6_ (*x*=0.0 to 1.0) did not lead to compounds with other Li/Sn ratios than that of the previously reported phases Li_5_SnP_3_,^[19]^ *α*‐ and *β*‐Li_8_SnP_4_ ^[8]^ and Li_14_SnP_6_.^[7]^ Interestingly, no ordered structure is observed for Li_5_SnP_3_ and Li_14_SnP_6_, whereas two polymorphs with distinctly ordered cation positions are found for the Li_8_SnP_4_. In addition, there is no evidence of a phase width of the compounds. The disorder in Li_5_SnP_3_ and Li_14_SnP_6_ is in accordance with the recorded ^31^P and ^119^Sn MAS NMR spectra, which exhibit extremely broad resonances. Such broad resonances hint for a vague chemical environment of the P and Sn atoms. Nevertheless, the chemical shift of the signals is within the range of the resonances reported for the ordered structures of *α*‐ and *β*‐Li_8_SnP_4_, indicating the presence of [SnP_4_] tetrahedra in Li_5_SnP_3_, which in accordance with the charge are expected to form edge‐sharing [Sn_2_P_6_]^10−^ dimers as found as ordered variant in Li_10_Si_2_P_6_.^[16]^

A two‐step synthesis route, including mechanical alloying and subsequent annealing of the samples, yields all four compounds in high purity and on a gram scale allowing for a profound determination of the properties.

The low ionic conductivity of *σ*_Li_=3.2(2) ⋅ 10^−7^ S cm^−1^ of Li_5_SnP_3_ in combination with vacant octahedral sites on the one hand, and the high ionic conductivity of *α*‐ and *β*‐Li_8_SnP_4_ as well as of Li_14_SnP_6_ with partially filled octahedral sites on the other unequivocally proof the importance of the participation of the octahedral voids in ion motion. In order to lower the activation energy one can either lower the energy barrier for Li^+^ motion between neighboring sites or raise the energy level of the respective sites. The partial occupation of the energetically unfavorable octahedral voids in the Li‐rich phosphidotetrelates corresponds to the latter case and leads to an overall flattening of the energy landscape. In this context the investigation of less promising Li^+^‐conducting materials with insufficient ionic conductivities for application, plays a key role in the understanding of the criteria to design and tailor next‐generation ionic conductors.

## Supporting Information Summary

Details of crystal structure determination of Li_5_SnP_3_, details on the investigation of the system Li_8−4*x*_Sn_1+*x*_P_4_ (*x*=−0.333 to +0.333), differential scanning calorimetry (DSC), ^6^Li, ^119^Sn, and ^31^P MAS NMR spectroscopy.

## Conflict of interest

The authors declare no conflict of interest.

1

## Supporting information

As a service to our authors and readers, this journal provides supporting information supplied by the authors. Such materials are peer reviewed and may be re‐organized for online delivery, but are not copy‐edited or typeset. Technical support issues arising from supporting information (other than missing files) should be addressed to the authors.

Supporting Information

## Data Availability

The data that support the findings of this study are available in the supplementary material of this article.
