# Supplementary material for: Li5SnP3 – a Member of the Series Li10+4x Sn2−x P6 for x=0 Comprising the Fast Lithium‐Ion Conductors Li8SnP4 (x=0.5) and Li14SnP6 (x=1)
Source: Chemistry. 2022 Jan 27;28(10):e202104219. doi: 10.1002/chem.202104219 (PMC9303179; doi:10.1002/chem.202104219)
Supplement: Supplementary file 1 — Supporting Information [file CHEM-28-0-s001.pdf]

Chemistry–A European Journal

Supporting Information

Li_5SnP_3 – a Member of the Series $Li_{10+4x}Sn_{2-x}P_6$ for $x = 0$ Comprising the Fast Lithium-Ion Conductors Li_8SnP_4 ($x = 0.5$) and $Li_{14}SnP_6$ ($x = 1$)

Stefan Strangmüller, David Müller, Gabriele Raudaschl-Sieber, Holger Kirchhain, Leo van Wüllen, and Thomas F. Fässler*

Content

Details of the crystal structure determination of Li_5SnP_3 S2

Details of the investigation of the system $Li_{10+4x}Sn_{2-x}P_6$ (x = 0.0 to 1.0) S8

Differential scanning calorimetry (DSC) S12

^{6}Li, ^{119}Sn and ^{31}P MAS NMR spectroscopy S14

References S17

Details of the crystal structure determination of Li_5SnP_3

Results of the crystal structure determination of Li_5SnP_3 from powder X-ray diffraction data at 293 K with fixed site occupancy factors (s.o.f.) according to the composition Li_5SnP_3

Table S1. Atomic coordinates of Li_5SnP_3 (Z = 1.33) at 293 K.

Atom	Wyckoff positions	*x*	*y*	*z*	s.o.f.
P	4*a*	0	0	0	
Sn	8*c*	1/4	1/4	1/4	0.167
Li	8*c*	1/4	1/4	1/4	0.833

Table S2. Anisotropic displacement parameters (Å^2) of Li_5SnP_3 (Z = 1.33) at 293 K.

atom	U_{11}	U_{22}	U_{33}	U_{23}	U_{13}	U_{12}
P	0.0132(5)	0.0132(5)	0.0132(5)	0	0	0
Sn	0.0139(5)	0.0139(5)	0.0139(5)	0	0	0
Li	0.0139(5)	0.0139(5)	0.0139(5)	0	0	0

Table S3. Selected interatomic distances in Li_5SnP_3 (Z = 1.33) at 293 K.

Atom pair			*d* / Å
P	Sn/Li	8×	2.5925(1)
Sn/Li	P	4×	2.5925(1)
	Sn/Li	6×	2.9936(1)

Results of the crystal structure determination of Li_5SnP_3 from powder X-ray diffraction data at 293 K with released site occupancy factors resulting in the composition $Li_{6.70(1)}Sn_{1.30(1)}P_4$

Table S4. Details of the Rietveld structure refinements of $Li_{6.70(1)}Sn_{1.30(1)}P_4$ (Z = 1) at 293 K.

Empirical formula	$Li_{6.70(1)}Sn_{1.30(1)}P_4$
T / K	293
Formula weight / g mol^{-1}	324.72
Space group (no.)	$Fm\bar{3}m$ (225)
unit cell parameters / Å	a = 5.98715(4)
Z	1
V / Å^3	214.615(3)
$\rho_{calc.}$ / g cm^{-3}	2.509
2θ range / deg	5.000-49.9441
R_p	3.87 %
R_{wp}	5.18 %
R_{exp}	3.87 %
X^2	1.79
GOF	1.3
R_{Bragg}	1.61 %
R_f	1.33 %
Depository no.	CSD-2074710

Table S5. Atomic coordinates of $Li_{6.70(1)}Sn_{1.30(1)}P_4$ (Z = 1) at 293 K.

Atom	Wyckoff positions	x	y	z	s.o.f.
P	4*a*	0	0	0	
Sn	8*c*	1/4	1/4	1/4	0.162(1)
Li	8*c*	1/4	1/4	1/4	0.838(1)

Table S6. Anisotropic displacement parameters (Å^2) of $Li_{6.70(1)}Sn_{1.30(1)}P_4$ (Z = 1) at 293 K.

Atom	U_{11}	U_{22}	U_{33}	U_{23}	U_{13}	U_{12}
P	0.0141(6)	0.0141(6)	0.0141(6)	0	0	0
Sn	0.0127(6)	0.0127(6)	0.0127(6)	0	0	0
Li	0.0127(6)	0.0127(6)	0.0127(6)	0	0	0

Table S7. Selected interatomic distances in $Li_{6.70(1)}Sn_{1.30(1)}P_4$ (Z = 1) at 293 K.

Atom pair			d / Å
P	Sn/Li	8×	2.5925(1)
Sn/Li	P	4×	2.5925(1)
	Sn/Li	6×	2.9936(1)

Results of the crystal structure determination of Li_5SnP_3 from single crystal X-ray diffraction data at 150 K with fixed site occupancy factors according to the composition Li_5SnP_3

Table S8. Atomic coordinates of Li_5SnP_3 (Z = 1.33) at 150 K.

Atom	Wyckoff positions	*x*	*y*	*z*	s.o.f.
P	4*a*	0	0	0	
Sn	8*c*	1/4	1/4	1/4	0.167
Li	8*c*	1/4	1/4	1/4	0.833

Table S9. Anisotropic displacement parameters (Å^2) of Li_5SnP_3 (Z = 1.33) at 150 K.

Atom	U_{11}	U_{22}	U_{33}	U_{23}	U_{13}	U_{12}
P	0.0083(2)	0.0083(2)	0.0083(2)	0	0	0
Sn	0.0089(2)	0.0089(2)	0.0089(2)	0	0	0
Li	0.0089(2)	0.0089(2)	0.0089(2)	0	0	0

Table S10. Selected interatomic distances in Li_5SnP_3 (Z = 1.33) at 150 K.

Atom pair			*d* / Å
P	Sn/Li	8×	2.5925(1)
Sn/Li	P	4×	2.5925(1)
	Sn/Li	6×	2.9936(1)

Results of the crystal structure determination of Li_5SnP_3 from powder X-ray diffraction data at 293 K with released site occupancy factors resulting in the composition $Li_{6.70(1)}Sn_{1.30(1)}P_4$

Table S11. Crystallographic data and refinement parameters of $Li_{5.06(1)}Sn_{0.95(1)}P_3$ ($Z = 1.33$) or $Li_{6.74(1)}Sn_{1.26(1)}P_4$ ($Z = 1$) at 150 K with released site occupancy factors.

Empirical formula	$Li_{5.06(1)}Sn_{0.95(1)}P_3$ / $Li_{6.74(1)}Sn_{1.26(1)}P_4$
Formula weight / g mol^{-1}	320.25
Crystal size / mm^3	0.08 × 0.08 × 0.09
Crystal color	black
T / K	150
Crystal system	cubic
Space group (no.)	$Fm\bar{3}m$ (225)
Unit cell parameters / Å	a = 5.9541(7)
Z	0.75 / 1
V / Å^3	211.08(7)
$\rho_{calc.}$ / g cm^{-3}	2.583
μ / mm^{-1}	4.644
F(000) / e	147
θ range / deg	5.934 – 46.355
Index range (hkl)	$-7 \leq h \leq 11$, $-10 \leq k \leq 11$, $-11 \leq l \leq 4$
Reflections collected	348
Independent reflections	72
R_{int}	0.0101
Reflections with $I > 2\sigma(I)$	72
Absorption correction	multi-scan
Data / restraints / parameters	72 / 0 / 5
Goodness-of-fit on F^2	1.235
R_1, wR_2 (all data)	0.0127, 0.0127
R_1, wR_2 [$I > 2\sigma(I)$]	0.0288, 0.0288
Largest diff. peak and hole (e Å^{-3})	0.201 / −0.252
Depository no.	CSD-2074709

Table S12. Atomic coordinates of $Li_{6.74(1)}Sn_{1.26(1)}P_4$ (Z = 1) at 150 K.

Atom	Wyckoff positions	*x*	*y*	*z*	s.o.f.
P	4*a*	0	0	0	
Sn	8*c*	1/4	1/4	1/4	0.1575(7)
Li	8*c*	1/4	1/4	1/4	0.8426(7)

Table S13. Anisotropic displacement parameters (Å^2) of $Li_{6.74(1)}Sn_{1.26(1)}P_4$ (Z = 1) at 150 K.

Atom	U_{11}	U_{22}	U_{33}	U_{23}	U_{13}	U_{12}
P	0094(1)	0094(1)	0094(1)	0	0	0
Sn	0.0088(1)	0.0088(1)	0.0088(1)	0	0	0
Li	0.0088(1)	0.0088(1)	0.0088(1)	0	0	0

Table S14. Selected interatomic distances in $Li_{6.74(1)}Sn_{1.26(1)}P_4$ (Z = 1) at 150 K.

Atom pair			*d* / Å
P	Sn/Li	8×	2.5782(2)
Sn/Li	P	4×	2.5782(2)
	Sn/Li	6×	2.9771(3)

Details of the investigation of the system $Li_{10+4x}Sn_{2-x}P_6$ (x = 0.0 to 1.0)

The PXRD patterns of the reactive mixtures as found after ball milling show only small deviations in terms of the intensities of the occurring phases (Figure S1a). Due to the mechanical alloying process the samples exhibit rather poor crystallinity and, thus, relatively weak and strongly broadened reflections. However, all mixtures show reflections of a cubic lattice comparable to that of Li_5SnP_3. In addition, all samples contain small amounts of remaining β-Sn. However, the compositions with a higher content of Sn also show a higher share of the residual element, whereas in the diffraction patterns corresponding to $x = 0.75$ and 1.0 small amounts of Li_3P are observed. The shift of the reflection occurring between 33.5 and 34.0 ° in Figure S1b indicates an increase of the cell parameter with increasing x or an increasing amount of Li. The determination of the exact cell parameters is not feasible.

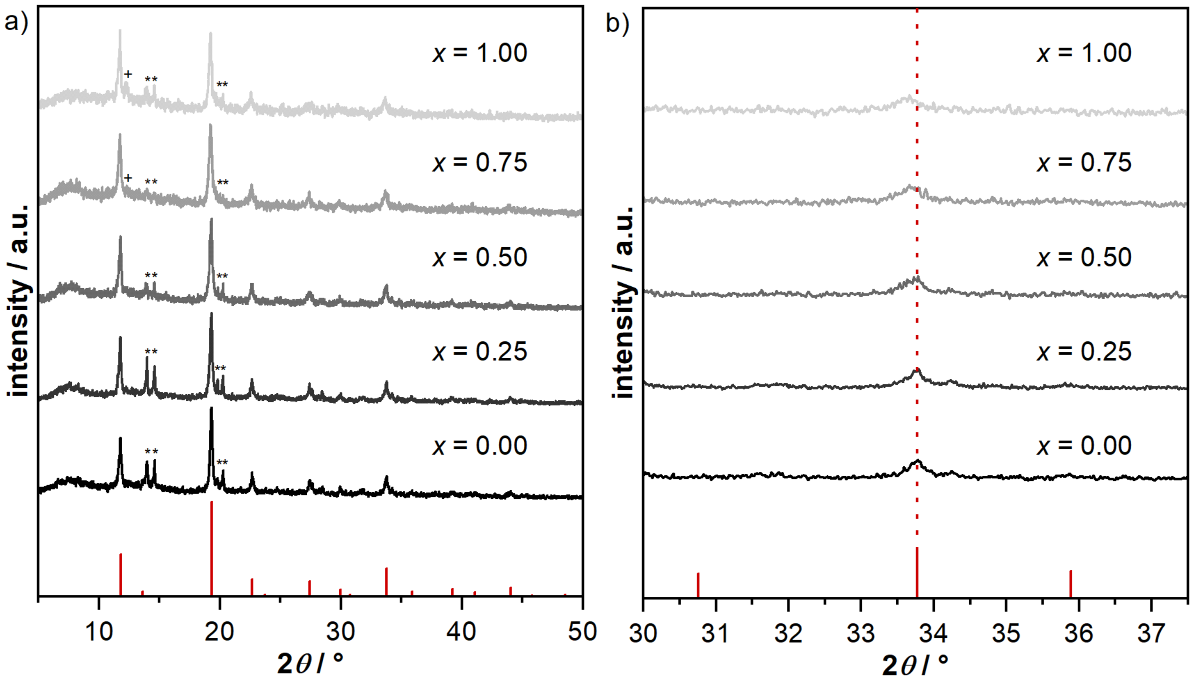

Figure S1. a) Powder X-ray diffraction patterns of the reactive mixtures with the stoichiometry $Li_{10+4x}Sn_{2-x}P_6$ (x = 0.0 to 1.0). The calculated diffraction pattern of Li_5SnP_3 is shown in red, reflections of the side products β-Sn and Li_3P are indicated by * and +, respectively. b) Magnification of the section between 30.0 and 37.5 ° indicating an increase of the cell parameter with increasing values for x.

The PXRD patterns after annealing at 673 K (Figure S2a) show the formation of Li_5SnP_3 (with marginal amounts of β-Sn as a side-phase) for $x = 0.0$ as well as phase-pure α-Li_8SnP_4 for $x = 0.5$. The patterns of all other compositions show mixtures of Li_5SnP_3 and α-Li_8SnP_4 and additional side-phases, such as β-Sn (Sn-rich) and Li_3P (Li-rich). The magnification of the patterns at higher angles also reveals changes of the cell parameter upon the formation of α-Li_8SnP_4 (Figure 2b).

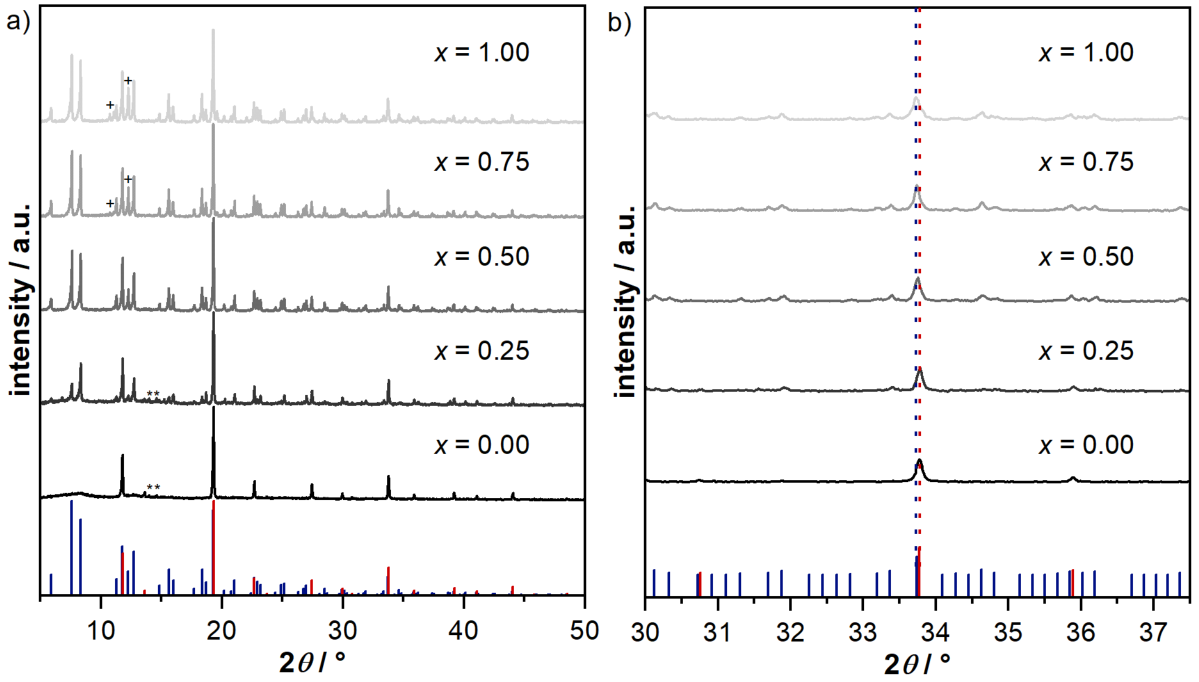

Figure S2. a) Powder X-ray diffraction patterns of the reactive mixture with the stoichiometry $Li_{10+4x}Sn_{2-x}P_6$ ($x = 0.0$ to 1.0) after annealing at 673 K. The calculated diffraction patterns of Li_5SnP_3 and α-Li_8SnP_4 are shown in red and blue, respectively. Reflections of the side products β-Sn and Li_3P are indicated by * and +, respectively. b) Magnification of the section between 30 and 37.5 ° shows the different cell parameters of Li_5SnP_3 and α-Li_8SnP_4.

The PXRD patterns after annealing at 773 K (Figure S3a) analogously show the formation of Li_5SnP_3 (with marginal amounts of β-Sn as a side-phase) for $x = 0.0$ as well as phase-pure β-Li_8SnP_4 for $x = 0.5$. A mixture of Li_5SnP_3 and β-Li_8SnP_4 together with small amounts of β-Sn is observed for $x = 0.25$, whereas the lithium-rich mixtures with $x = 0.75$ and 1.0 resulted in the formation of a mixture of β-Li_8SnP_4 and $Li_{14}SnP_6$ (with small amounts of Li_3P) and almost phase-pure $Li_{14}SnP_6$, respectively. The magnification of the patterns at higher angles shows the expected increase of the cell parameter corresponding to the formation of Li_5SnP_3, β-Li_8SnP_4 and $Li_{14}SnP_6$ (Figure 3b).

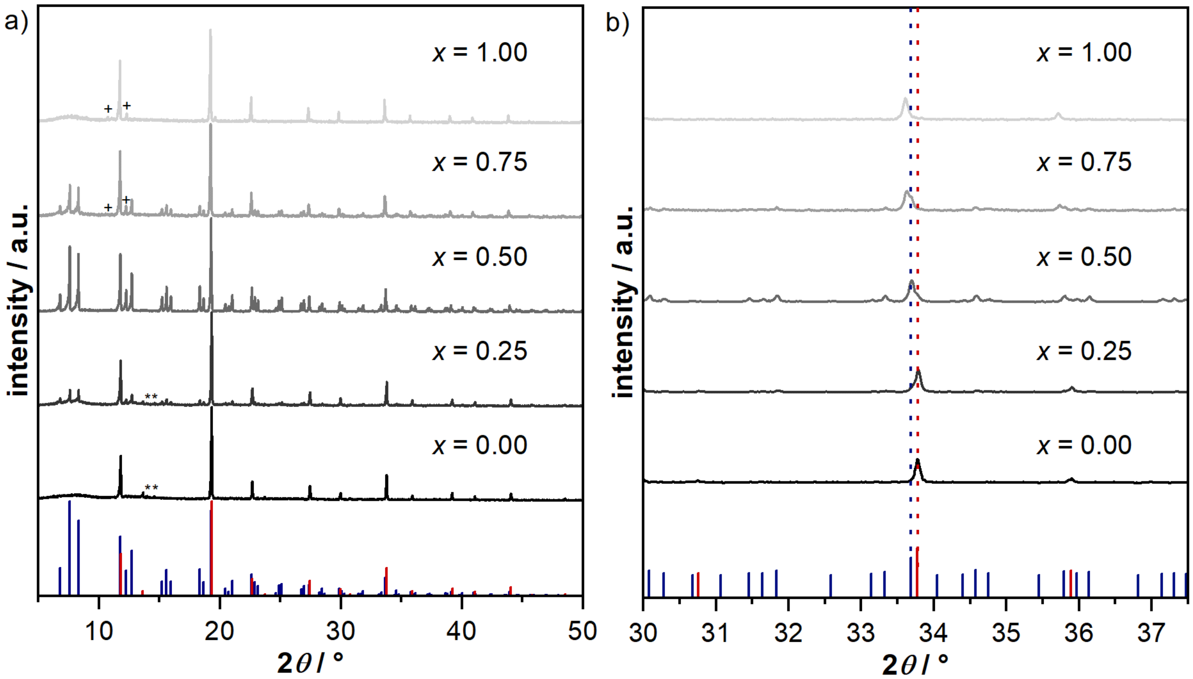

Figure S3. a) Powder X-ray diffraction patterns of the reactive mixture with the stoichiometry $Li_{10+4x}Sn_{2-x}P_6$ ($x = 0.0$ to 1.0) after annealing at 773 K. The calculated diffraction patterns of Li_5SnP_3 and β-Li_8SnP_4 are shown in red and blue, respectively. Reflections of the side-products β-Sn and Li_3P are indicated by * and +, respectively. b) Magnification of the section between 30 and 37.5 ° indicates the different cell parameters of Li_5SnP_3 and β-Li_8SnP_4.

The PXRD patterns after annealing at 973 K (Figure S4a) are dominated by the reflections of $Li_{14}SnP_6$ (Li-rich) and Li_5SnP_3 (Sn-rich) with β-Li_8SnP_4, β-Sn and Li_3P as side-phases in agreement with the total composition. Magnification of the patterns at higher angles allows for a rough estimation of the share of Li_5SnP_3, β-Li_8SnP_4 and $Li_{14}SnP_6$ within the product (Figure 4b).

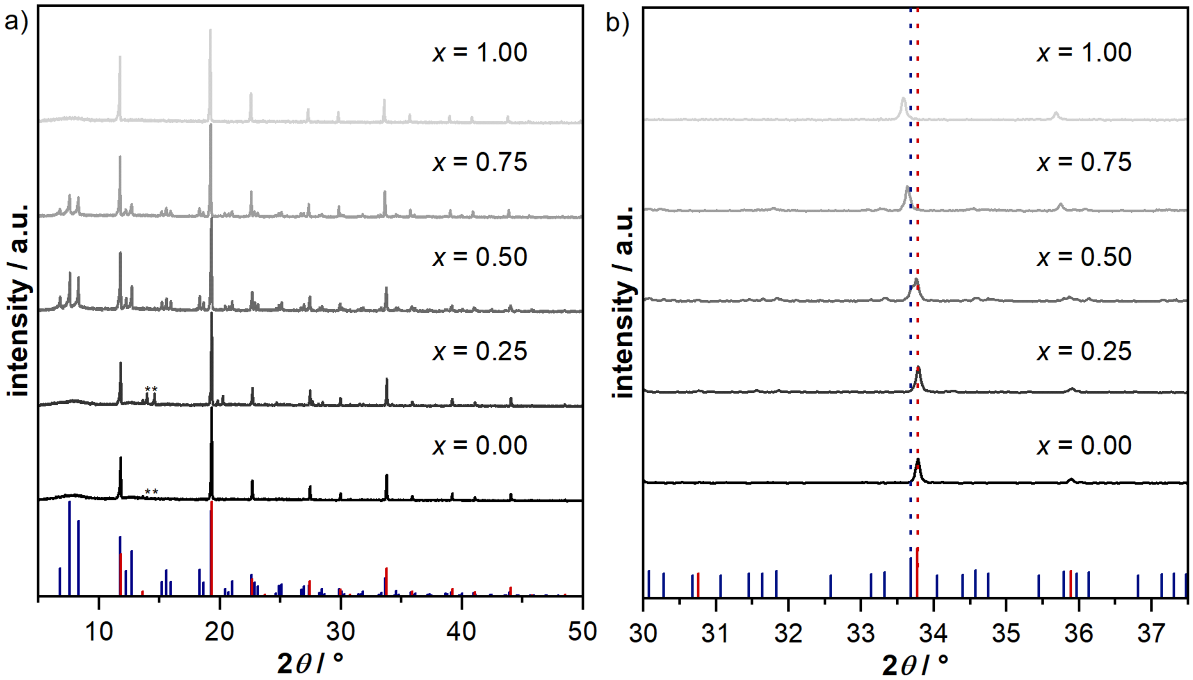

Figure S4. a) Powder X-ray diffraction patterns of the reactive mixtures with the stoichiometry $Li_{10+4x}Sn_{2-x}P_6$ (x = 0.0 to 1.0) after annealing at 973 K. The calculated diffraction patterns of Li_5SnP_3 and β-Li_8SnP_4 are shown in red and blue, respectively. Reflections of the side-products β-Sn are indicated by *. b) Magnification of the section between 30 and 37.5 ° indicates the different cell parameters of Li_5SnP_3, β-Li_8SnP_4 and $Li_{14}SnP_6$.

Differential scanning calorimetry (DSC)

The recorded thermograms of the reactive mixture "Li_5SnP_3" and the corresponding crystalline phase are shown in Figure 5.

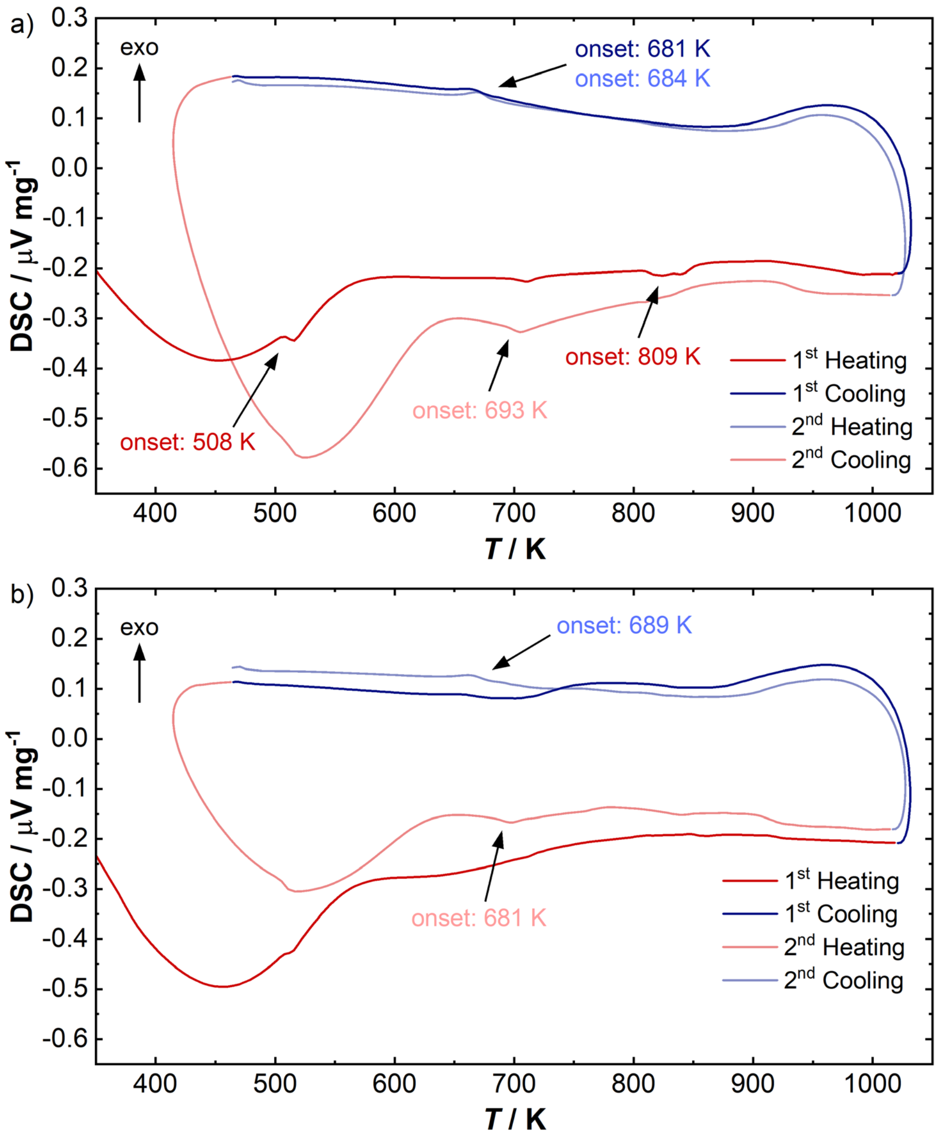

Figure S5. a) DSC thermogram of the reactive mixture "Li_5SnP_3". b) DSC thermogram of Li_5SnP_3. The arrows and numbers indicate the onset temperatures of the corresponding thermal effects.

The first thermal effect observed at an onset temperature of 508 K represents the melting point of elemental Sn (*β*-Sn) which is also observed as a side-phase after the preparation of the reactive mixture *via* mechanical alloying. During the following heating and cooling cycles one reversible and one irreversible effect appear at an onset temperature of about 690 and 810 K, respectively. The signals can be referred to (partially occurring) order-disorder transitions as the PXRD pattern of the sample after the DSC measurement indicates the occurrence of additional, slightly broadened reflections probably assignable to a superstructure comparable to *α*- or *β*-Li_8SnP_4 (Figure 6a).
The thermogram as well as the PXRD pattern of the crystalline phase are almost identical to the corresponding data of the reactive mixture. Since the amount of *β*-Sn is increased after the measurement it can be assumed that Li_5SnP_3 (partially) decomposes at high temperatures resulting in a mixture of *β*-Sn and a compound that is closely related to *α*- or *β*-Li_8SnP_4 as indicated by certain superstructure reflections (Figure 6b).

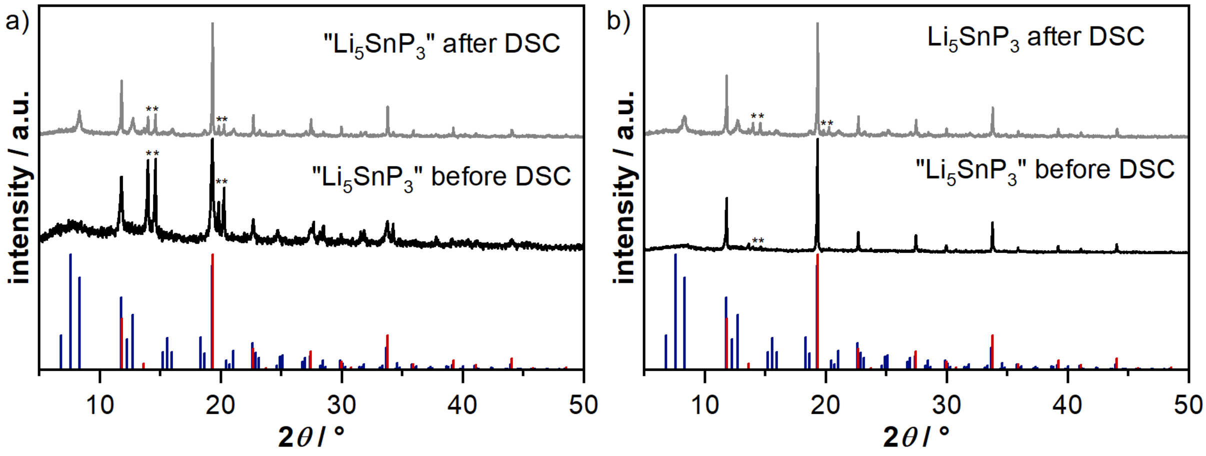

Figure S6. a) Powder X-ray diffractogram of the reactive mixture "Li_5SnP_3" before and after DSC measurement. b) Powder X-ray diffractogram of Li_5SnP_3 before and after DSC measurement. The calculated diffraction pattern of Li_5SnP_3 is shown in red, and *β*-Sn is indicated by *.

^{6}Li, ^{119}Sn and ^{31}P MAS NMR spectroscopy

^{6}Li MAS NMR Spectroscopy

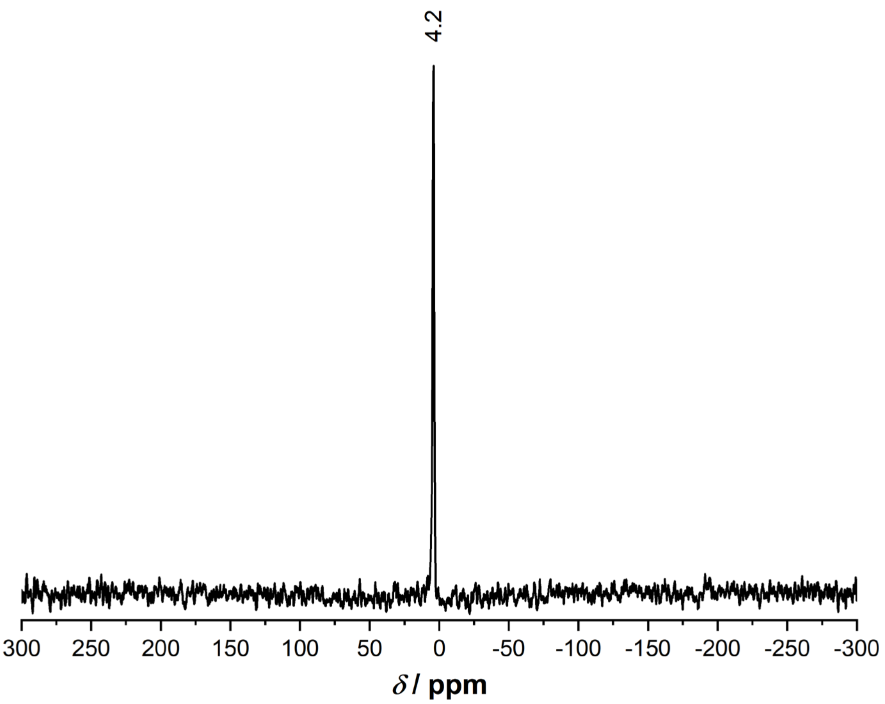

Figure S7. ^{6}Li MAS NMR spectrum of Li_5SnP_3 (15 kHz).

^{31}P MAS NMR spectroscopy

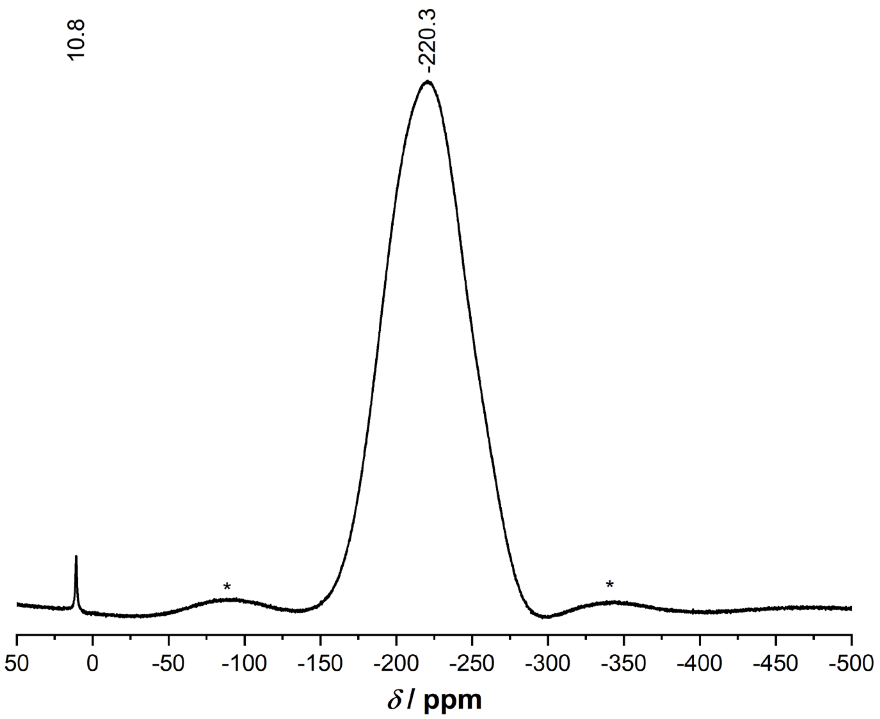

Figure S8. ^{31}P MAS NMR spectrum of Li_5SnP_3 (15 kHz). Spinning sidebands are indicated by *. The resonance at a chemical shift of 10.8 ppm reveals the formation of very small amounts of phosphates during data collection.[1-3]

^{119}Sn MAS NMR spectroscopy

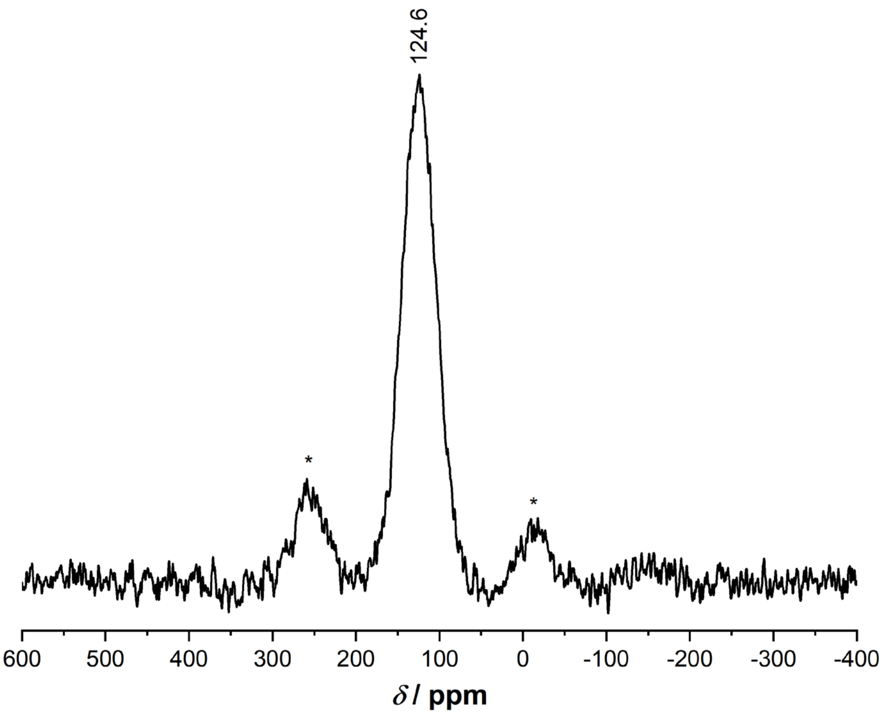

Figure S9. ^{119}Sn MAS NMR spectrum of Li_5SnP_3 (15 kHz). Spinning sidebands are indicated by *.

References

[1] R. J. Kirkpatrick, R. K. Brow, *Solid State Nucl. Magn. Reson.* **1995**, *5*, 9-21.

[2] R. K. Brow, D. R. Tallant, S. T. Myers, C. C. Phifer, *J. Non-Cryst. Solids* **1995**, *191*, 45-55.

[3] Y. Deng, C. Eames, J.-N. Chotard, F. Lalère, V. Seznec, S. Emge, O. Pecher, C. P. Grey, C. Masquelier, M. S. Islam, *J. Am. Chem. Soc.* **2015**, *137*, 9136-9145.
